# NDDN: A Cloud-Based Neuroinformation Database for Developing Neuronal Networks

**DOI:** 10.1155/2018/3839094

**Published:** 2018-07-03

**Authors:** Jiangbo Pu, Xiangning Li

**Affiliations:** ^1^Institute of Biomedical Engineering, Chinese Academy of Medical Sciences and Peking Union Medical College, Tianjin 300192, China; ^2^Britton Chance Center for Biomedical Photonics, Wuhan National Laboratory for Optoelectronics-Huazhong University of Science and Technology, Wuhan, Hubei 430074, China; ^3^MoE Key Laboratory for Biomedical Photonics, Collaborative Innovation Center for Biomedical Engineering, School of Engineering Sciences, Huazhong University of Science and Technology, Wuhan, Hubei 430074, China; ^4^HUST-Suzhou Institute for Brainsmatics, Suzhou 215125, China

## Abstract

Electrical activity of developing dissociated neuronal networks is of immense significance for understanding the general properties of neural information processing and storage. In addition, the complexity and diversity of network activity patterns make them ideal candidates for developing novel computational models and evaluating algorithms. However, there are rare databases which focus on the changing network dynamics during development. Here, we describe the design and implementation of Neuroinformation Database for Developing Networks (NDDN), a repository for electrophysiological data collected from long-term cultured hippocampal networks. The NDDN contains over 15 terabytes of multielectrode array data consisting of 25,380 items collected from 105 culture batches. Metadata including culturing and recording information and stimulation/drug application protocols are linked to each data item. A Matlab toolbox named MEAKit is also provided with the NDDN to ease the analysis of downloaded data items. We expect that NDDN may contribute to both the fields of experimental and computational neuroscience.

## 1. Introduction

Spontaneous neural activity plays a critical role in the development and function of the nervous system [1–6]. The diversity and the complexity of neural activity patterns are believed to be the key to understand how the neuronal network operates whether it is in or out of the normal state [7–16]. Investigations on the characteristics of spontaneous activity, stimuli-evoked response, and drug-mediated activity are amongst the fundamentals of neuroscience research. Further, computational biologists and researchers in the field of computer science are also inspired by the dedicated organization of structures and functions of neuronal networks [12, 13, 17–20]. It is found that developing neuronal networks share similar and critical aspects with other biological networks, the global interconnected computer networks (the Internet), social networks, and even the universe [17, 19, 21–26]. Gathering information on the evolving dynamics of developing neuronal networks is therefore attracting increasingly attentions [11, 27].

As an important *in vitro* model of the nervous system, dissociated neuronal networks have been used by neuroscientists since over 30 years ago. But until the recent decade, it is difficult to perform long-term observation of networkwide activities from the cultured neurons and manipulation of the network dynamics without impairing the health of the culture [11, 20, 28]. Growing on the multielectrode arrays, large random neuronal networks developing *in vitro* are demonstrated to display general properties of neural systems and enables extensive measurements and manipulations of neuronal dynamics with little interference with the network state [10, 28–30]. However, although many databases have been built to promote genetic/proteomic researches and investigations of EEGs and ECGs [31, 32], there is still lack of a comprehensive database focused on collecting and sharing electrophysiological activities of long-term developing neuronal networks. Scattered in laboratories around the globe, it is difficult and inconvenient to access and utilize such information.

In the present study, we describe how we construct the NDDN (Neuroinformation Database for Developing Networks), a new repository for electrophysiological data acquired from long-term cultured hippocampal networks and the features of both the NDDN and the data it stores.

## 2. Materials and Methods

### 2.1. Dissociated Hippocampal Cultures

**Figure 1:**
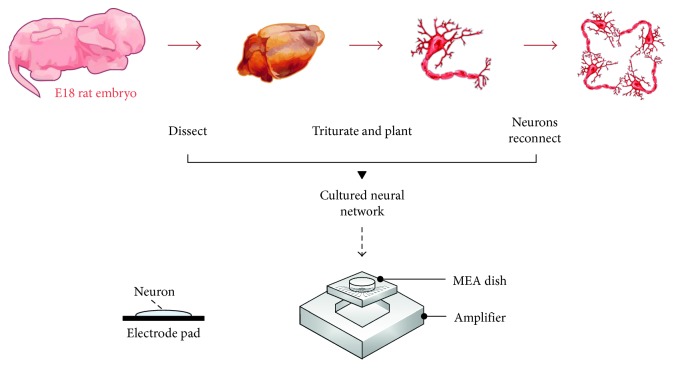
Preparation of dissociated hippocampal networks with multielectrode arrays.

**Figure 2:**
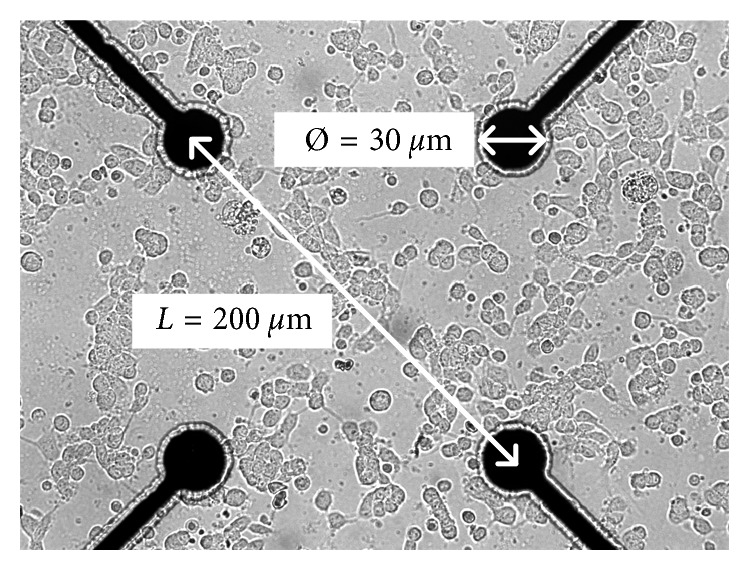
The neurons on a multielectrode array at day 3.

All experimental procedures used in this study were approved by the Animal Ethics Committee of Huazhong University of Science and Technology. As described previously [8, 33, 34], the hippocampus was extracted quickly from E18-19 Wistar rat embryos and then was gently dissected and dissociated by trypsin (Sigma, 10 min at 37°C). The hippocampal neurons were plated onto a culture dish with an embedded multielectrode array (MEA, Ayanda Biosystems SA, Lausane, Swiss; Multichannel Systems, Reutlingen, Germany) at a density of 2500 cells/mm^2^ (Figures 1 and 2). To improve cell adhesion, poly-L-lysine was used to coat the array before seeding. The culture medium contained 1 ml neurobasal medium (Invitrogen) with B27 supplement (Invitrogen), 10% equine serum (HyClone), and 0.5 mM Glutamax (Invitrogen). Half of the medium was replaced every 2 days. The dishes were placed in a 37°C, 5% CO_2_ water jacketed incubator.

### 2.2. Data Collection and Organization

Raw data were collected using an MEA1060 recording system (Multichannel Systems, Reutlingen, Germany). Each dish contains 59 recording electrodes. Extracellular signals were continuously sampled at 25–50 kHz. We used a threshold method (5 × standard deviation) to convert the raw data stream into a spike train. Raw data were saved into ∗.mcd format by MC_Rack (Multichannel Systems, Reutlingen, Germany) and could be later converted into hierarchical data format (HDF) for sharing. The ∗.mcd file also can be read into Matlab using Neuroshare interface (http://neuroshare.sourceforge.net/) with the provided toolbox. Spike data were saved into ∗.mat format with Matlab structures. All data files were uploaded into the distributed storage of the server, and the path to each data file in the file system was saved as a property of the corresponding data item in the database.

Metadata (refer to Table 1 for detailed information) which contain the experimental details for each data file are organized as experimental sets rather than individual items. To facilitate data management, metadata items can be tagged as a different category by users; therefore, items from the same experiment or with identical experimental protocol, condition, and parameters can be grouped into a working set. If available, the microscopy images of corresponding cultured network were collected and linked to the metadata item.

### 2.3. Database Design and Implementation

**Figure 3:**
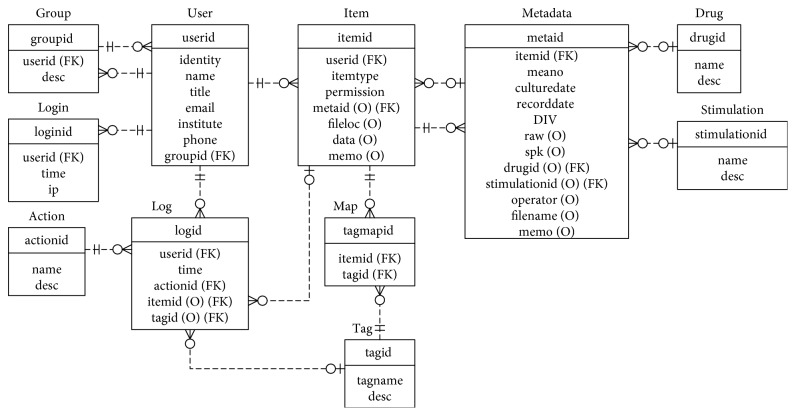
The entity-relationship data model of the NDDN. Relationships among tables are shown with dotted lines. Entities are shown using boxed frames with their names labeled above. Primary keys are shown in the first row of the entity box. Foreign keys are labeled with (FK). The relationships between each table are shown with the dashed links between boxed frames.

We used the MySQL relational database management system to store and perform data queries. Eleven tables were created to provide indexed and structured data organization, as well as secured data access. The entity-relationship (ER) data model representing the relationships among these tables is shown in Figure 3. The core table is named as “item.” Identified by a unique ID, each item in the database has a record in the “item” table which stores essential information of the item, such as the owner, the type, and access permission, and can be further linked to detailed experimental descriptions. If the item is a data file, then we save its path as “fileloc” in the item record. If the item is a photo or a piece of code, the data are saved directly in the item table as “data.” Related metadata information is linked to the table named “Metadata,” and the tagging information is stored in the “Tag” table which is linked to the main item table by the “Map.”

The NDDN is designed to have the access control for each group of users. Users do not have to save their passwords into the database. Based on the OpenID framework, the authentication is accomplished by third-party providers, such as Google accounts and Microsoft accounts service. The returned OpenID identity is saved into the database and later used to identify the user. Therefore, NDDN users can login into the system with their Gmail, Hotmail accounts, or other credentials that support the OpenID communication protocol. In the database, each data item has an “owner” property, and the access permission may be granted to three classes of users: the item owner, the group that the owner belongs, and to all users.  Table 2 shows the UNIX-like permission code string for data items. Additionally, to provide detailed information of data manipulation, login actions and data access history are saved into the database.

### 2.4. Service-Based Web Portal

The core functions of the NDDN have been implemented as RESTful (representational state transfer) web services with PHP. Based on the MVC (model-view-controller) coding pattern, resources in the database were able to reach via URIs, for example, “http://bmp.hust.edu.cn/neuro_db/v2/data/618352/retrieve.” Data access may require a token which was returned after the user was authenticated. After data query, structured information will be returned in XML format, and the raw/spike data will be returned in binary form.

### 2.5. Customized Scripts and Data Visualization

The NDDN has implemented a Python interface using interprocess communication (IPC) mechanism. With preinstalled SciPy package (http://www.scipy.org/), users can upload their scripts and perform various computing tasks using NDDN data. Considering security issues, access to the file system and other critical system resources in Python is limited. The visualization of NDDN data items were also implemented using SciPy and Matplotlib (http://matplotlib.org/). Graphics are generated at the back-end and then displayed at the front-end afterwards. In the NDDN , all scripts including visualization and user-uploaded algorithms written in Python are also saved as data items with specific item types. The access permission rules are identical to other regular items.

## 3. Results and Discussion

### 3.1. Database Content

In the current release, 25,380 items of multielectrode recording data (15 terabytes+) are collected from 105 culture batches. 5,233 items of experiments involved in stimulation protocols (single-site stimulation, paired stimulation, multisite stimulation, one-polar/bipolar configurations, etc.). 2,599 items of experiments involved in drug testing protocols ((2R)-amino-5-phosphonovaleric acid (APV), bicuculline (BIC), 6-cyano-7-nitroquinoxaline-2,3-dione (CNQX), tetrodotoxin (TTX), octanol (OCT), carbenoxolone (CBX), brain-derived neurotrophic factor (BDNF), etc.). Recordings which were taken with stimulations and/or drug applications were grouped with spontaneous recordings before and after the application, which helps users to perform quantitative analysis focused on whether or how the network dynamics was affected by a specific protocol.

**Figure 4:**
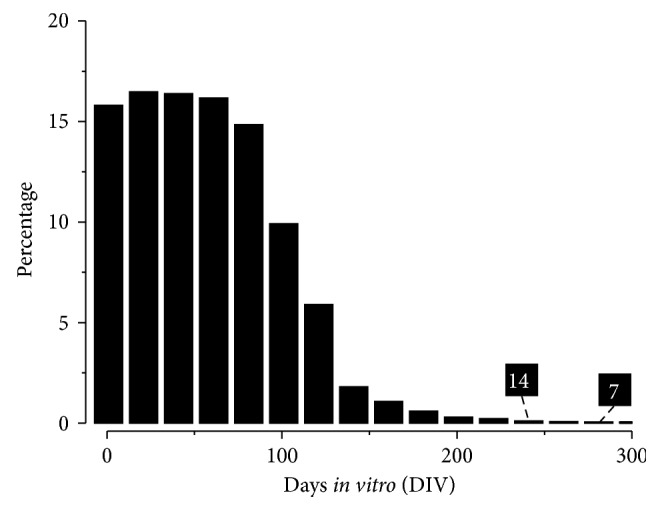
The distribution of DIVs of data items in NDDN.

As a database for developing neuronal networks, the diversity of spontaneous recordings from varied developmental ages of cultured neuronal networks is an important index. The distribution of recording dates (also known as, days *in vitro* (DIV)) of existing data items is shown in Figure 4 which is also regularly undated in the statistics page of the NDDN. Abundant scientific experiments have been conducted with cultured neuronal networks between 1 and 9 weeks [5, 7–9, 11, 28, 30]. In NDDN, most recordings fell within the similar time range, providing a rich repertoire of data resources for neuroinformatics and modeling researches. Further, we have data from 15+ culture batches which lived over 150 DIVs. These data sets of long-term developing networks are believed to benefit the understanding of evolving dynamics of neuronal networks during *in vitro* development [5].

The histogram shows the distribution of DIVs of NDDN items (in percentage). Note that most recordings were taken before 100 DIVs. Although long-term cultures were rare, NDDN still has recordings between 200 and 300 DIVs. Numbers in black boxes show the actual numbers of items in the corresponding DIV range.

### 3.2. Website Interface and NDDN Web Services

**Figure 5:**
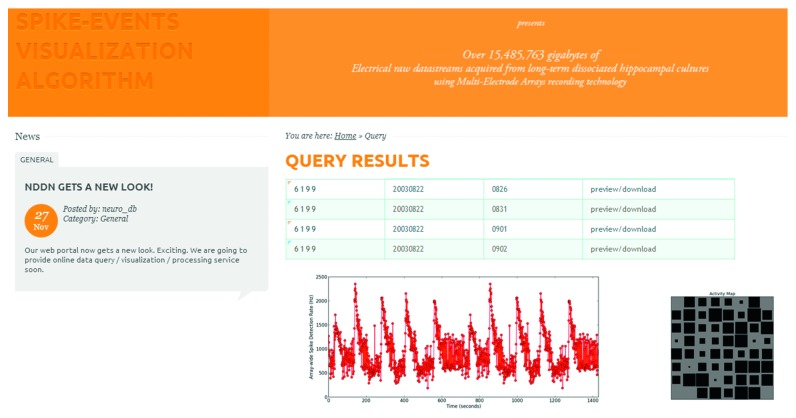
A screenshot of an example page of data query results.

Users can access the NDDN at http://bmp.hust.edu.cn/neuro_db/. Pages of introductory materials and related publications can be browsed without login. To access pages of data query and download requires educational user authentication (currently, the website is hosted on a university server (Intel Xeon E3 CPUs with 64 Gigabytes RAM) in the China Education and Research Network (CERNET) which is required by our university but may block some international access due to government policy; we are trying to apply for a permission to deploy the database in a public cloud run by a private company). An example page of data query and built-in visualization using the provided Matlab toolbox is shown in Figure 5.

Example query results are shown in the table. The output of built-in visualization functions is shown below. Array-wide spike detection rate is shown with the line graph with red dots. The channel activity hot map is shown next to the line graph.

The NDDN accepts various search conditions: culture date, recording date, culture dish number, DIV, stimulation protocol, drug name, and the operator who conducted the experiment. As mentioned in Methods, tags can be labeled on individual data items. Users can put the same tag on all the items in the returned search result, which saves the search results for reuse in the future. For example, if one had performed a specific search based on last returned search results or with multiple conditions, then she/he could directly load the results by the tag next time. Besides, users can specify whether their own tags will be exposed to all users, which helps to keep the database organized and encourages constructive sharing.

The core functions of the NDDN are exposed as web service APIs (application programming interfaces).  Table 3 shows the list of core APIs. Each request contains all of the necessary information to accomplish the request. The client does not need to hold any session state. User authentication information will be sent to the client after successful login and will be used as a token for next calls. The stateless RESTful web service APIs helps researchers to develop tools that can directly download/upload data from/to the NDDN.

### 3.3. Matlab Toolbox

**Figure 6:**
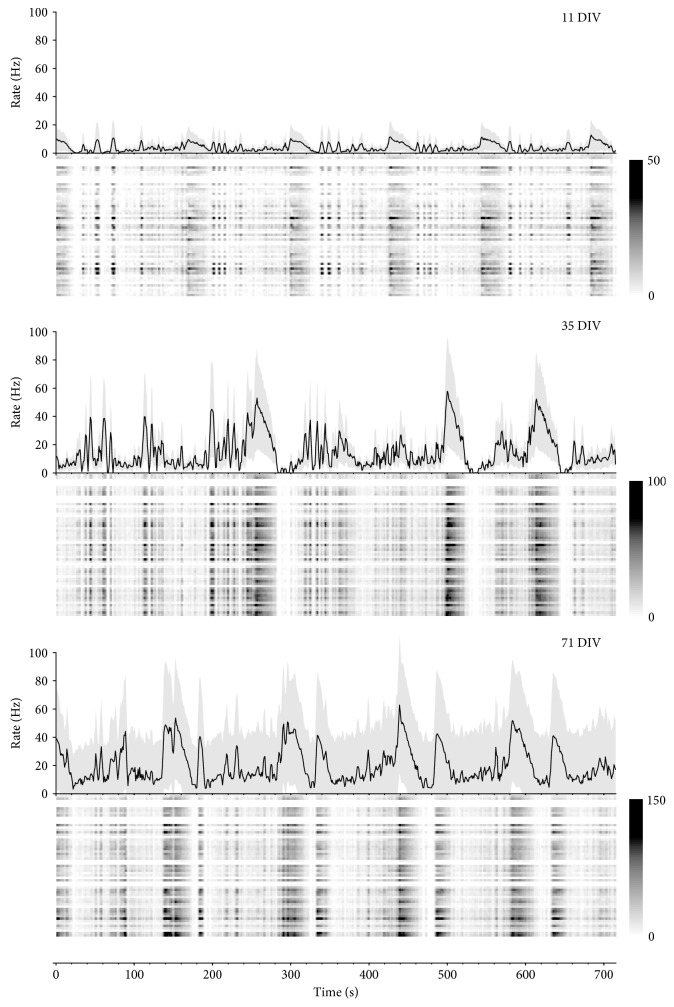
The diversity of activity patterns of developing neuronal networks. In each subplot, the upper panel shows array-wide spike detection rates per second at different ages of the same culture (DIVs are labeled in the top right corner). The spike detection rates of individual electrodes in the array are shown with raster plots in grayscale (rows: electrodes). Color bars indicate spike rates (Hz). Shades in the line graphs show standard deviations of rates among channels of the corresponding array.

The NDDN also provides a toolbox written in Matlab code to reduce the difficulty in using NDDN data. The MEAKit (multielectrode array ToolKIT) toolbox is freely available at the NDDN website. Users can also contribute to the future development of the toolbox by submitting bugs and issues or even committing their codes to make their own version branch at the GitHub portal (https://github.com/pujb/meakit). The key functions are listed in Table 4. Briefly, M-functions in the MEAKit toolbox are grouped into different categories by their purposes. Data files can be loaded into Matlab workspace by I/O functions, for example, util_load_mcds(). We have already implemented multiple built-in functions in the “Calculation” directory to perform some commonly used classical neural dynamics analyses [30], as well as some newly adopted algorithms, such as neuronal avalanche analysis and fractal quantification [5]. Users can use their own way to visualize the results or they can use functions in the “Plot” directory to generate graphs that meet the common publishing standards of scientific journals.  Figure 6 shows changing firing patterns of an example neuronal network during development. Scripts which were written for specific purposes are located in the “Scripts” directory. For detailed information, see the toolbox references topics in the “Help” directory.

### 3.4. Comparison with Other Databases

Compared to a rich repertoire of online bioinformatics databases, there are much fewer databases aimed at providing electrophysiological information of neuronal networks, let alone databases that specially focused on multielectrode array data of *in vitro* developing networks. The CARMEN project (http://www.carmen.org.uk/) provides a powerful international cooperation framework for sharing codes, data, and models of multiple levels of the neural system [27]. The Allen Brain Atlas (ABA) (http://www.brain-map.org/) and the Visible Brain-wide Networks (VBN) project (http://vbn.hust.edu.cn/) are famous for their precious image resources and powerful tools [35]. Among these databases, the CARMEN project aims at providing multiple types of data (including electrophysiological data and images) from various sources at different levels of the neural system (from the cellular level to the whole-brain level). The CARMEN project is powerful for its virtual laboratory framework for enabling whole brain leveldata but also online exploitation of neurophysiological data and online code running and analyzing. The Allen Brain Atlas and the VBN project aim at providing cell type databases, toolboxes, and detailed images of brain connectivity. Currently, there is little report of a database focused on dissociated cultured neuronal networks. Clearly, the dissociated neuronal networks lack many features of the intact whole brain, but the essential nature of the neural cells and the network formed by the neurons and other neural cells are kept in these dissociated cultures. Therefore, observing the neurons and how they form and develop into a network may help us to better understand the mechanism of the brain. Also, many detailed and dissected analyses of neural circuits are not feasible in living animals and humans. Here, we collected our dissociated neuronal network data by multielectrode arrays (MEAs). Spontaneous activities and activities under stimulated and medicated conditions were recorded. Although there are obvious limitations in the NDDN for its limited data sources and data types, we are trying to release the unique data of dissociated cultured neuronal networks as a specialized database tailored for developing neuronal networks on MEAs. The NDDN provides a large set of unique data which is exclusive at the moment as far as we know and the developmental information for cultured neuronal networks which is unique currently.

## 4. Conclusions

Electrophysiological activity patterns in developing neuronal networks are of great importance in the fundamental research of neural dynamics and neural coding. Here, we described a new database for developing networks, which has over 15 terabytes data at present. The NDDN can be utilized by computational neuroscientists and modelers to extract the characteristics and derive new models, shedding new light on novel algorithm development and evaluation. Experimental neuroscientists may be also benefitted by NDDN which can be seen as a database containing preliminary trials with various experimental protocols. We expect that the NDDN will better serve the researchers in the related field as a basis for insight into the neural dynamics at the network level.

## Figures and Tables

**Table 1:** Metadata descriptions.

Property	Description	Type
MEA number	The number of each MEA culture dish	Char
Culture date	The date when neurons were seeded onto the MEA dish	Date time
Recording date	The date when recording was made	Date time
DIV (days *in vitro*)	The age of the culture	Integer
Is Raw	True = raw data	Boolean
Is Spk	True = spike data	Boolean
Drug ID	If available, specifies the name of the drug applied to the culture	Foreign key
Stimulation ID	If available, specifies the name of the stimulation protocol	Foreign key
Operator	Specifies the person who conducted the experiment	Char
Filename	Original filename	Char
Memo	Other descriptions	Char

**Table 2:** Permission codes of data items.

Roles	Owner	Users in the same group	All users
Permission code	RWDX|----|----	----|RWDX|----	----|----|RWDX

R: read; W: write; D: delete; X: execute.

**Table 3:** The NDDN web service APIs.

Format	Function
Siteroot/data/[ID]	Retrieves metadata of the specified item
Siteroot/data/[ID]/get	Downloads the item
Siteroot/data/[ID]/delete	Deletes the item
Siteroot/sti_protocol/[ID]	Retrieves the specified stimulation protocol
Siteroot/drug_protocol/[ID]	Retrieves the specified drug protocol
Siteroot/[userID]/auth	Performs user authentication
Siteroot/[userID]/logout	Logout the user

**Table 4:** MEAKit functions categories.

Category	Description
Calculation	Implemented algorithms (e.g., interspike interval, array-wide spike detection rate, spike sorting, connectivity analysis, and neuronal avalanche analysis)
Common	Commonly used subfunctions
Conversion	Unit conversion and data type conversions
Help	Help documents
IO	File access functions
Model	Computational models
Plot	Visualization functions
Scripts	Customized M-scripts for specified data analysis
Other boxes	Included third-party toolboxes and libraries
